# Scar masses from granulation tissue

**Published:** 2016

**Authors:** Asad Khan, Sandro Lanzon-Miler, Kamran Rostami

**Affiliations:** *Department of Gastroenterology, Milton Keynes University Hospital United Kingdom *

**Question**

A 50 years old Male known with a diagnosis of ulcerative colitis since October 2012 (Montreal Class E3) was seen for further follow up in gastroenterology outpatient. He was on Meselazine 2.4 gm per day. The last flare up he had was 2.5 years ago in April 2013 when he was treated successfully with a course of oral steroids. His colitis has been in a stable state with no further flare ups ever since. During his follow-up in Gastroenterology clinic in Sept 2015, he mentioned that he is opening his bowels 4-5/day with a consistency between watery to loose stool that was “normal” for him. He denied having significant bleeding or cramps. He also denied having a family history for colorectal cancer (CRC) or polyps. Physical examination was unremarkable. Blood test revealed a normal CRP of 7, FBC, LFTs and U &E were also normal. Further colonoscopy surveillance were organised for assessment.

**What is the diagnosis? What is the prognosis and appropriate management?**

**Figure 1:**
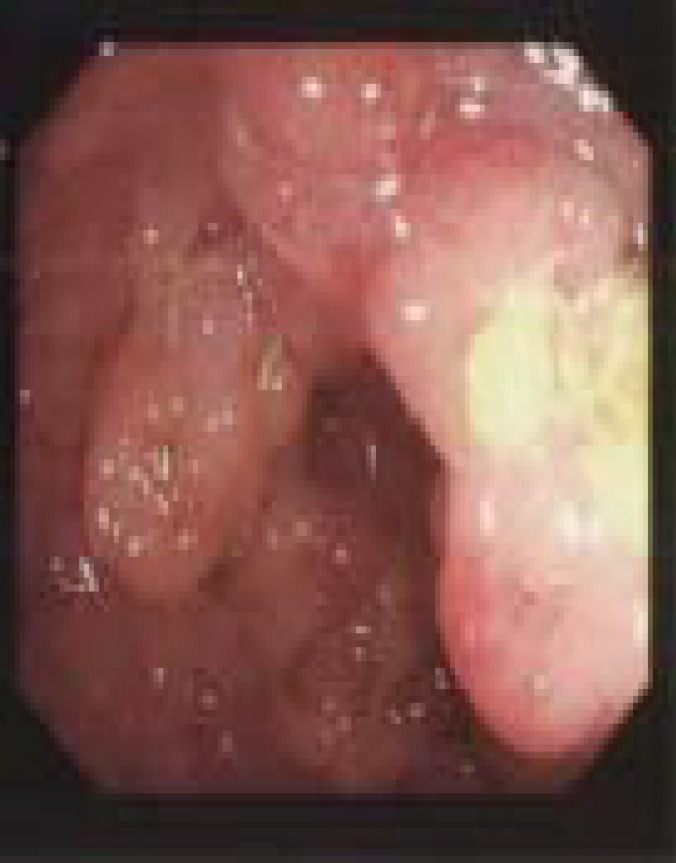
Colonoscopy; distal ascending colon. The colonoscopist reported that the ascending abnormalities caused stenosis but just enough open to pass the scope getting through to the Caecum

 **Answer**

Giant Inflammatory Polyps (GIPs) in IBD.

**Figure:**
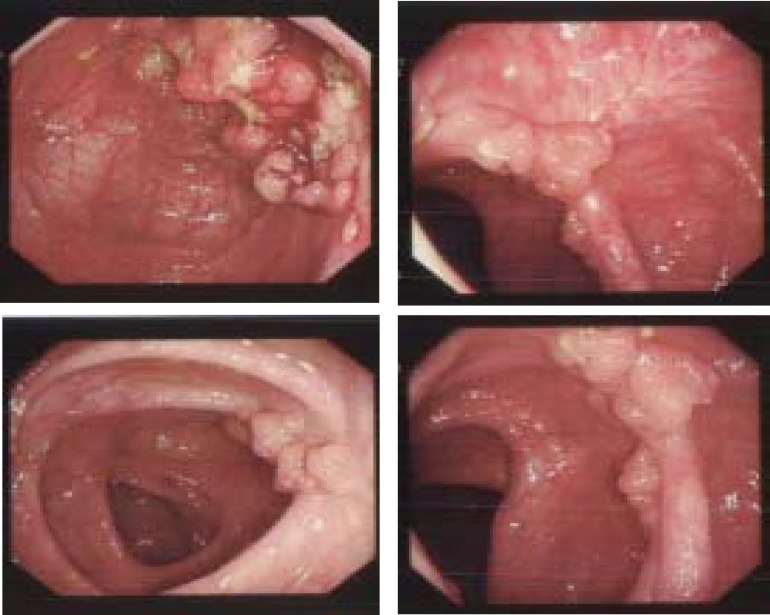


## Discussion

Giant inflammatory polyps are uncommon with a reported prevalence of 4.6% and two thirds of cases being associated with Crohn’s disease (1,2).

Inflammatory polyps are not exclusive to inflammatory bowel disease and can occur in infectious, ischemic colitis, borders of ulcers as well as mucosal anastomosis (3).

It is not exactly clear why these polyps form with some studies showing enhanced de novo synthesis of all types of collagen, in patients with ulcerative colitis, as well as increased expression of collagenases (4,5).

Giant inflammatory polyps are defined as inflammatory polyps more than 1.5cm (1).

Most are asymptomatic although there could be symptoms of underlying IBD. GIPs are known to cause obstruction, protein-losing enteropathy, anaemia and bleeding (6-8). GIPs are generally deemed benign but there have been case reports of dysplasia and malignancy within these polyps (9,10). Despite their benign nature, the presence of inflammatory polyps have been demonstrated to be associated with an increased risk of malignancy (11,12). Based on increased risk for malignant transformation BSG guidelines, for colorectal cancer screening, recommend escalation of risk category to “intermediate” and performing 3 yearly surveillance colonoscopy rather than every 5 years (13).

Microscopically these show inflammatory infiltrates overlying the muscularis mucosae with deep fissure-like ulcers, features of chronic mucosal inflammation with lymphoid and nerve hyperplasia in the surrounding mucosa (6-8, 14-17).

The pathologist reported inflammatory polyps might show features of crypt distortion, cryptitis, crypt abscesses, loss of muscularis mucosae, sumucosal fibrosis, and Paneth cell hyperplasia.

In almost all of the cases, these are not amenable to medical management but some case reports have demonstrated regression of these lesions after medical management (2, 18, 19). BSG guideline states that prophylactic colectomy should be discussed with patient especially if colonoscopist feels that the value of surveillance is compromised (13). Our patient was discussed in lower GI MDT and MDT recommended referral to Oxford team for second opinion.
